# RNA-seq provides insights into potato deubiquitinase responses to drought stress in seedling stage

**DOI:** 10.3389/fpls.2023.1268448

**Published:** 2023-09-14

**Authors:** Xun Tang, Fujun Sun, Ning Zhang, Birendra Bahadur Rana, Raju Kharel, Pan Luo, Huaijun Si

**Affiliations:** ^1^State Key Laboratory of Aridland Crop Science, Gansu Agricultural University, Lanzhou, China; ^2^College of Life Science and Technology, Gansu Agricultural University, Lanzhou, China; ^3^Nepal Agricultural Research Council, National Potato Research Program, Lalitpur, Nepal; ^4^Department of Genetics and Plant Breeding, Agricultural and Forestry University, Chitwan, Nepal

**Keywords:** deubiquitination, deubiquitinase, genome-wide analysis, abiotic stress, RNA-Seq, *Solanum tuberosum* L

## Abstract

Ubiquitination is a specific protein degradation and reversible post-translational modification process that can be reversed by deubiquitinase (DUBs). DUBs can hydrolyze and release ubiquitin in the substrate protein so that the substrate can avoid degradation or change its activity, and it has an impact on plant growth and development, cell cycle, abiotic stress response, and other biological processes. Transcript sequences of potato varieties “DM1-3”, “Atlantic” and “Cooperation-88” downloaded from Potato Genome Resources were used for genome-wide identification of the DUB gene family using Hidden Markov Models and verified in the NCBI CD-Search tool. The characteristics of DUB genes from different potato varieties were analyzed including subcellular localization, gene structural motifs, phylogenetic tree, and sequence homology. Polyethylene glycol 6000 (PEG6000) induced drought stress transcriptome analysis was performed on the “Atlantic”, and differentially expressed genes were screened, with emphasis on the characterization of deubiquitinase. DUB genes have a complex gene structure, often with a large number of exons and alternative splicing. Their promoters contain abundant abiotic stress-responsive elements, such as 425 MYC, 325 ABRE, and 320 MYB. There are also a large number of orthologous genes in the DUBs of the three potato varieties, and these genes are often clustered in similar regions on the genome. We performed transcriptome sequencing of the potato under PEG-induced drought stress and analyzed it for the first time using the Atlantic as a reference genome. We identified a total of 6067 down-regulated differentially expressed genes (DEGs) and 4950 up-regulated DEGs under PEG-induced drought stress. We screened the expression of DUBs and observed that 120 DUBs were up-regulated where most of them functioned in the nucleus, and the interacting proteins of DUBs were also localized in the nucleus. We have comprehensively identified and analyzed potato DUBs, and the accurately aligned transcriptome data which will further deepen the understanding of DUBs involved in the regulation of osmotic stress.

## 1 Introduction

Increased global warming has resulted in frequent extreme weather events, which have seriously affected crop growth and yields, thus posing a major threat to agricultural food production and food security (Duchenne-Moutien and Neetoo, 2021). Potato (*Solanum tuberosum* L.) is widely grown due to its universal adaptability, ease of cultivation, and high nutritional value, making it the third-largest food crop in the world (http://www.fao.org/). It plays an important role in world food security, especially in developing countries (Camire et al., 2009; Hoopes et al., 2022). But potatoes are susceptible to environmental factors, especially in the early stages of growth, including drought, salt, and extreme temperatures (Dahal et al., 2019). Among them, drought is the most important factor causing food loss, it is more than all the combined yield loss by pathogens. Demand for water for agriculture is increasing due to population growth, while the availability of fresh water for agriculture is decreasing due to global climate change (Gupta et al., 2020). Breeding and selection processes are important in improving the stress resistance of potatoes to increase the yield. Screening potato genes resistant to abiotic stress, and then breeding varieties resistant to abiotic stress is an effective way to achieve this goal (Kikuchi et al., 2015; Campbell et al., 2021).

The ubiquitination pathway is an important post-translational modification, and its members are involved in the regulation of plant growth and development and response to abiotic stresses (Stone, 2019; Xu and Xue, 2019). Proteins are ubiquitinated by three sequential steps involving a cascade of three enzymes: E1 ubiquitin-activating enzyme, E2 ubiquitin-conjugating enzyme, and E3 ubiquitin ligase (Glickman and Ciechanover, 2002). The number of ubiquitin molecules and the type of ubiquitin linkages determine the fate of ubiquitinated substrate proteins (Komander and Rape, 2012; Nakagawa and Nakayama, 2015). Ubiquitination and deubiquitination coordinate the binding of ubiquitin to its substrate proteins. This makes the activation and recycling of ubiquitin molecules by DUBs essential to maintain the reversibility and balance of ubiquitination modifications (Mevissen and Komander, 2017). Most DUBs are cysteine proteases that rely on active centers composed of cysteine, histidine, and aspartic acid to catalyze isopeptidase bonds between polyubiquitin units (Kouranti et al., 2010). The former of these classes are further divided into six protein families, including ubiquitin carboxyl-terminal hydrolase (UCH), ubiquitin-specific protease (USP), ovarian tumor proteases (OTU), Machado–Joseph domain (MJD), JAB1/MPN/MOV34 proteases (JAMM), and motif interacting with Ub-containing novel DUB family (MINDY), based on sequence conservation and domain structure. All DUBs utilize a catalytic triplet consisting of an active-site cysteine residue, histidine, and asparagine (aspartic acid. to catalyze the hydrolysis of ubiquitin bonds (Mevissen and Komander, 2017).

An increasing number of studies on DUBs in model plants such as *Arabidopsis thaliana* and rice (*Oryza sativa* L.) have shown that DUBs are important for plant growth, development, and stress resistance (Huang et al., 2017; Zhou et al., 2017; Ma et al., 2019; Shi et al., 2019). In this study, genome-wide identification and extraction of the DUB family in potatoes is studied for the first time. We have previously identified members of potato ubiquitin-conjugating enzyme (UBC) (Liu et al., 2019), U-box ubiquitin ligase (Tang et al., 2022), and small ubiquitin-like modifier (SUMO) (Ghimire et al., 2020), and elucidated that several of these genes respond to drought stress (Qi et al., 2020; Tang et al., 2020). In this study, deubiquitination enzyme genes of the classic variety “DM1-3” and two recently published cultivars “Atlantic” and “Cooperation-88” were identified and “Atlantic” expression profiles under PEG stress were analyzed by RNA-Seq. The DUB genes responding to PEG stress were screened out, which laid a good foundation for the subsequent exploration of deubiquitination regulation of drought response.

## 2 Materials and methods

### 2.1 Genome-wide identification and extraction of potato DUB gene family

The protein sequence FASTA files of the doubled monoploid *Solanum tuberosum* L. Group Phureja clone DM1-3 v6.1 (Pham et al., 2020), tetraploid Atlantic (Hoopes et al., 2022) and Cooperation-88 (C88). (Bao et al., 2022) were downloaded from the Spud DB database (http://spuddb.uga.edu/). Hidden Markov Model (HMM) files were accessed and downloaded from the Pfam database (http://pfam.xfam.org) which includes PF01398 (JAMM), PF02099 (MJD), PF02338 and PF10275 (OTU), PF01088 (UCH), PF00443 (USP), PF04424 (MINDY). The DUB family genes of potatoes were searched according to HMM files in hmmer 3.3.2 software. The retrieved genes were validated for conserved domains in the NCBI online tool Batch CD-Search, thereby identifying the potato DUB genes.

### 2.2 StDUBs feature analysis

The location and gene structure information of StDUBs were obtained from the Spud DB database, and the chromosomal location map and gene structure map was drawn using TBtools software. The physicochemical properties of StDUBs were analyzed using the online tool ProtParam (https://www.sib.swiss/) from the ExPASy website (Duvaud et al., 2021), and the subcellular localization of StDUBs was retrieved using the online tool WoLF PSORT (https://wolfpsort.hgc.jp) (Horton et al., 2007). Motifs of StDUBs proteins were analyzed using the online tool STREME (https://meme-suite.org/meme/tools/streme) (Bailey, 2021). The StDUBs gene structure annotation file (GFF3) was obtained from Spud DB, and the conserved motif in the promoter (upstream 2000bp sequence of the CDS sequence) was extracted. *Cis*-acting elements related to abiotic stress were, and then use the R language to draw statistical results. The protein sequences of AtDUBs from the TAIR database (https://www.arabidopsis.org/) were downloaded. The ClusterW tool in MEGA 11 software was used to perform multiple sequence alignment of the protein sequences of UCH subfamily genes in StDUBs and AtDUBs. The parameters were set to default values, and MEGA 11 software was used to construct a phylogenetic tree by the maximum likelihood method. Bootstrap value was set to 1000 AND other parameters to were set to default. The ggtree package of R language was used to optimize the evolutionary tree. The CDS sequences of StDUBs and AtDUBs were subjected to orthologous analysis in the seqkit tool and visualized and plotted genome collinearity by Python.

### 2.3 Plant material treatment and RNA-seq analysis

The shoot tips of potato tetraploid cultivar ‘Atlantic’ were soaked in 75% ethanol for 3 min and 3% NaClO for 10 min, then the tips were rinsed with sterile distilled water five times. Then the cleaned tips were transplanted in 3% sucrose and 0.7% NaClO Agar pH 5.8 in Murashige-Skoog (MS) medium and placed in a growth chamber with a 16/8 h light-dark cycle at a temperature of 23 ± 1)°C. After 3 weeks of growth, the single node with one leaf was cut off and transplanted into a liquid MS medium. When the plant height reached 10cm, it was randomly divided into a control group and a treatment group. The treatment group was replaced with MS+20% PEG6000 medium, and the control group was replaced with MS medium. All potato plants were collected after 12 hours, washed three times with distilled water, and stored in liquid nitrogen for analysis.

Transcriptome sequencing was performed at Shanghai Applied Protein Technology Co., Ltd. (Shanghai, China). Whole potato tissue culture seedlings were ground to powder under liquid nitrogen freezing. RNA was extracted using the RNAprep Pure Plant Kit (Tiangen, China), and RNA integrity was measured using an Agilent 4150. Paired-end libraries were prepared using the ABclonal mRNA-seq Lib Prep Kit (ABclonal, China), and PCR products were purified and library quality assessed using an Agilent Bioanalyzer 4150. Finally, sequencing was performed using the Illumina Novaseq 6000 sequencing platform.

### 2.4 Genome alignment and identification of DEGs

The clean reads were aligned with the Atlantic genome in the Spud DB database in HISAT2 software (http://daehwankimlab.github.io/hisat2/), and gene annotation information was extracted. The number of reads per gene was calculated using FeatureCounts (http://subread.sourceforge.net/), and the FPKM value was calculated based on the length of the transcript. Differential expression analysis of genes between groups was performed using DESeq2 (http://bioconductor.org/packages/release/bioc/html/DESeq2.html) with screening thresholds set as |log_2_FC|>1 and Padj <0.05 (Love et al., 2014).

### 2.5 StDUBs gene ontology and KEGG pathway analysis

GO feature enrichment analysis was performed using the clusterProfiler R package. The number of StDUBs contained in each GO item was counted, and the significance of StDUBs enrichment in each GO item was calculated (P<0.05). All GO terms were classified using three distinct dimensions, including biological process (BP), molecular function (MF), and cellular component (CC). KOBAS software was used to test the statistical enrichment of differentially expressed genes in the KEGG pathway of StDUBs and assigned them to different biological metabolic pathways, including cellular process environmental information processing, genetic information processing, metabolism, and organic systems. In STRING v11.5 (https://cn.string-db.org/cgi/input.pl), using potato as a model, hidden Markov clustering was used to predict protein-protein interactions.

### 2.6 qRT-PCR analysis

The potato variety “Atlantic” virus-free tuber was planted in a pot and placed in a greenhouse to grow. On the 30th day, it was randomly divided into two groups with similar plant height. The control group was watered once every 3 days, and the drought group was not watered for 12 consecutive days. The roots and leaves were ground in liquid nitrogen, and RNA was extracted by RNAprep Pure Plant Plus Kit (Tiangen, Beijing). Reverse transcription reaction was performed by FastKing RT Kit (Tiangen, Beijing), and fluorescence quantitative analysis was performed by SuperReal PreMix Plus (Tiangen, Beijing). Three biological and technical replications were performed for each treatment. We selected the 6 genes with the most significant differences in the RNA seq analysis (**Table S9**), and used the stable expression of *ef1α* (Tang et al., 2017) identified by us as the reference gene to analyze the DUB gene expression in potato under drought stress by 2^−ΔΔCT^ method.

## 3 Results

### 3.1 Identification and chromosomal location of StDUBs

**Figure 1:**
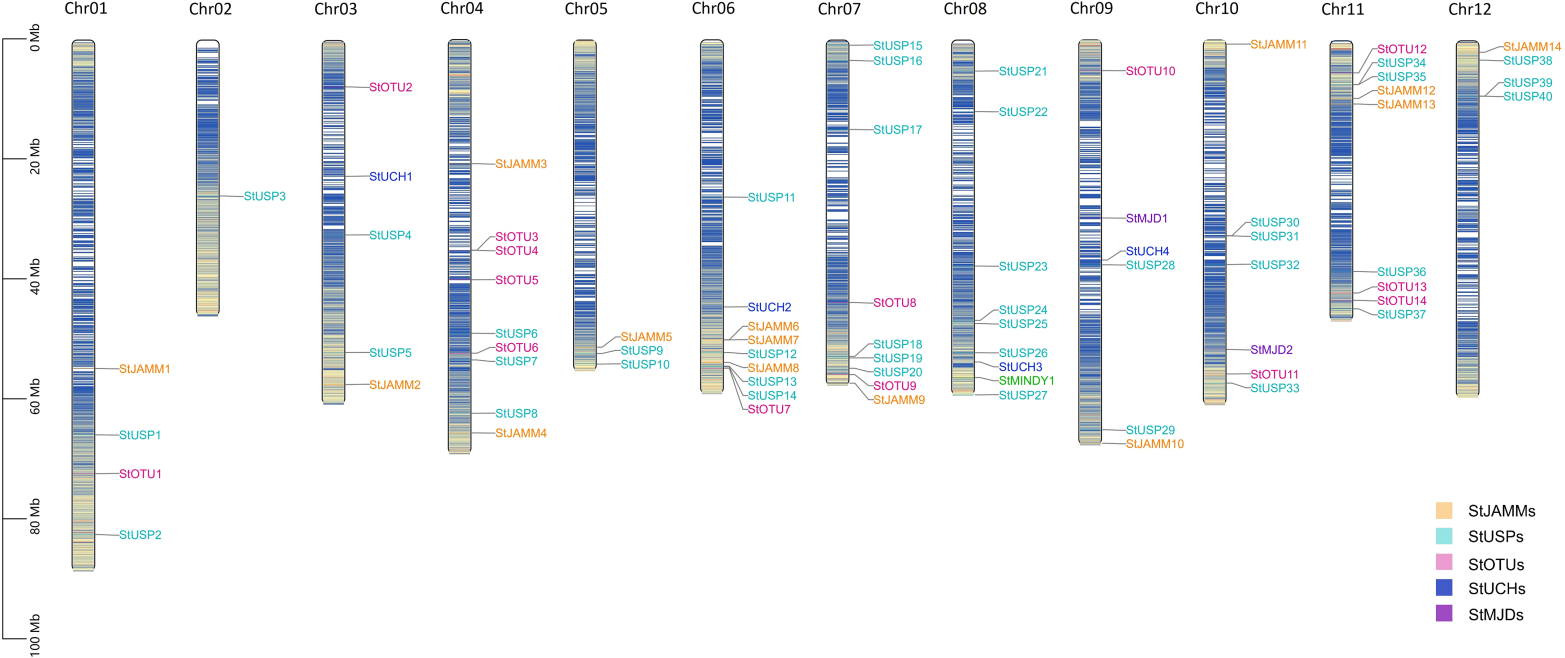
Chromosome mapping of DUB family genes in DM potato. Genes are named according to the sequence of genes on chromosomes.

Through hidden Markov search and NCBI Batch CD-Search conserved domain analysis, 75, 117, and 302 DUBs were identified in DM, Atlantic, and C88, respectively. They were all divided into six subfamilies: JAMM, MJD, OTU, UCH, USP, and MINDY. USP has the highest number of members in 40 (DM), 113 (Atlantic), and 169 (C88), and MINDY has the least members at 1 (DM), 3 (Atlantic), and 4 (C88) (**Tables 1**, **S1**). We named DM StDUBs according to their chromosomal locations and the subfamily of deubiquitinases, and used TBtools software to draw a chromosomal location map (**Figure 1**). StDUBs were asymmetric on chromosomes, with some chromosomes having a higher number of StDUBs, such as chromosomes 4 and 11, and others having a lower number, such as chromosomes 1 and 2. But there is a common phenomenon, that DUBs of the same type were localized in similar positions in large numbers which probably have a large number of replications in the evolutionary process.

**Table 1:** Number of ubiquitinase gene and transcript in potato.

Groups	DM		Atlantic		C88	
	Gene number	Transcript number	Gene number	Transcript number	Gene number	Transcript number
JAMM	14	23	38	63	50	78
MJD	2	2	4	4	8	8
OTU	14	27	32	83	54	178
UCH	4	9	10	17	17	45
USP	40	55	113	169	169	301
MINDY	1	1	3	5	4	9
Total	75	117	200	341	302	619

JAMM,JAB1/MPN/MOV34 proteases; MJD, Machado–Joseph domain; OTU, ovarian tumor proteases; UCH, ubiquitin carboxyl-terminal hydrolase; USP, ubiquitin-specific protease; MINDY, motif interacting with Ub-containing novel DUB family.

### 3.2 Structural features of StDUBs

**Figure 2:**
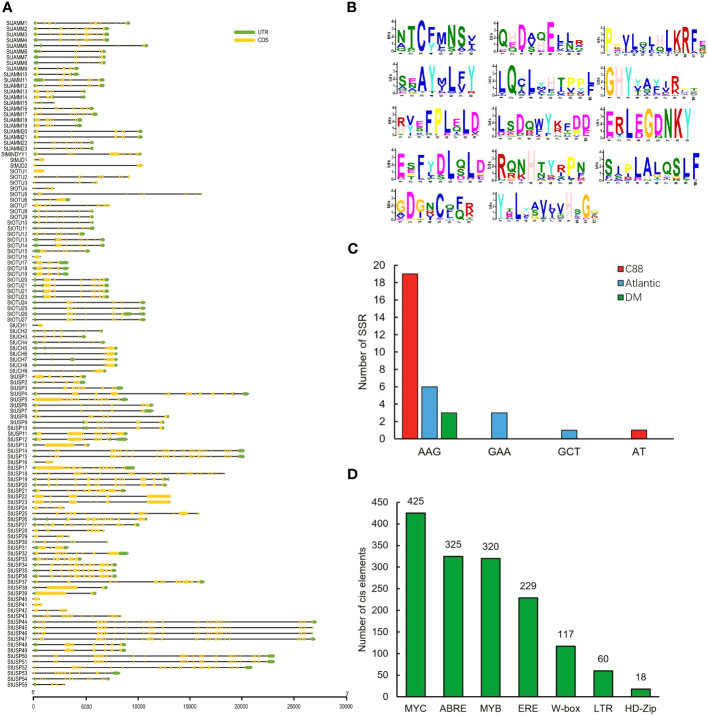
Information on potato deubiquitinase gene. **(A)** Structure of the DUBs gene of potato DM. **(B)** Conserved motifs of DUBs genes in potato DM. **(C)** SSRs of potato DUBs genes. **(D)** Homeopathic elements of DUBs genes in potato DM in response to abiotic stress (Hwarari et al., 2022).

We analyzed the structural characteristics of StDUBs (DM) using Tbtools software (**Figure 2A**). Except for 3 DUB genes with only one exon, the other DUBs are all discontinuous genes (DM), but different subfamilies showed differences in exon number. The members of the MJD subfamily contain only 2 exons, while the members of the USP subfamily generally contain a large number of exons. Among them, *StUSP44*, *StUSP45*, *StUSP46*, *StUSP47*, *StUSP50* and *StUSP51* consisted more than 32 exons that heralds the evolutionary complexity of these genes. The STREME tool was used to characterize the motifs of StDUBs (DMs). StDUBs (DM) contain 14 typical motifs with a length of 8–12 amino acids, and these motifs usually contain a large number of polar amino acids, and cysteine, which is the active center of deubiquitinase occurs in multiple motifs (**Figure 2B**). There were four kinds of SSRs; AAG, GAA, GCT, and AT, in potato DUBs, among which AAG was present in the largest number. C88 had the most number of SSRs among the three varieties, which was related to the number of DUBs (**Figure 2C**). StDUBs (DM) proteins vary in size, ranging from 120 to 1638 amino acids, members of the USP subfamily tend to be larger than other subfamilies, and all StDUBs are hydrophilic (**Table S2**). WoLF PSORT online software was used to analyze the subcellular localization of StDUBs and to score their likely localization. The vast majority of StDUBs localize to the nucleus, cytoplasm, and chloroplast, which corresponds to their function. Most of the members of the OTU and UCH subfamily are located in the chloroplast and cytoplasm respectively (**Tables 2**, **S3**). The PlantCare online tool was used for the analysis of *cis*-acting elements in the region 2000 bp upstream of the start codon of StDUBs (DM), and we found a large number of *cis*-acting elements associated with abiotic stress (**Figure 2D** and **Table S4**). Many of these are *cis*-acting elements recognized by ABA signaling associated transcription factors ABRE, MYB, and MYC. They have been shown to correlate with drought response.ethylene is another hormone that enhances plant drought resistance, and ethylene responsive element (ERE) has also been found in large quantities in potato DUBs.

**Table 2:** Quantitative distribution of subcellular localization of potato deubiquitinases.

Cellular structure	DM	Atlantic	C88
Chloroplast	21	57	90
Cytoskeleton	1	2	1
Cytoplasm	41	95	154
External complex	0	0	2
Golgi complex	0	5	3
Mitochondria	2	4	13
Nucleus	54	181	371
Peroxisomes	1	4	4
Plastids	3	7	13
Vacuoles	2	7	17

### 3.3 Phylogenetic and collinear analysis

**Figure 3:**
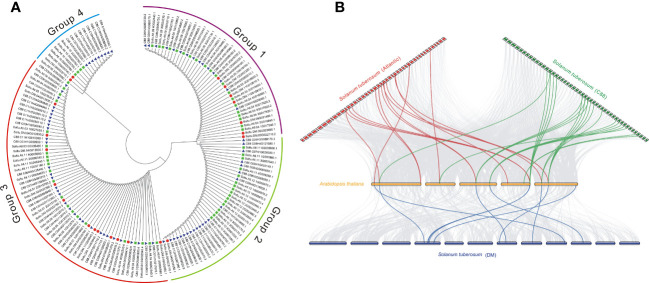
Phylogenetic and collinear analysis of potato DUBs. **(A)** Phylogenetic tree analysis of the potato JAMM subfamily. **(B)** Collinearity analysis of potato and *Arabidopsis* DUBs.

The phylogenetic trees of different subfamilies of StDUBs were constructed using MEGA 11. The JAMM family genes are mainly divided into four groups. Compared with other DUB subfamilies, JAMM subfamily gene members have clearer grouping. The homology of clustered genes decreased sequentially, which indicated that these genes had a common origin, and they were formed by homologous expansion during evolution. Group 2 contained 40 members, but only one was from DM, showing a large expansion of DUBs in tetraploid potato genes (**Figures 3A**, **S1**). The homology of the three cultivars of potato StDUBs with *Arabidopsis* and constructed a comparative collinearity relationship. The potato DUB family of DM, Atlantic, and C88 cultivars showed a high homology relationship with *Arabidopsis thaliana* genes, with 61, 184, and 171 pairs of orthologous pairs, respectively (**Figure 3B** and **Table S5**).

### 3.4 Transcriptome data and mapping statistics

This study compared the transcriptomic expression profile of potatoes under PEG stress. A total of 43.83 Gb of Clean bases were obtained from 6 potato tissue culture samples, each sample contained more than 43.27 million clean reads, and the Q30 quality scores of each sample were more than 89.22%. The correlation coefficients of samples within and between groups were calculated according to the FPKM of all genes in each sample, and the results showed that the correlation coefficients of samples within all groups were greater than 0.92 (**Figure S2A**). We performed principal component analysis (PCA), and the results showed that the samples within the group were highly clustered together (**Figure S2B**), suggesting reliable repeatability for each treatment. We searched clean reads in the DM v6.1 and Atlantic genome databases. It should be noted here that the Atlantic database was published only recently (Hoopes et al., 2022). The search results show that the comparison rate of each sample with Atlantic is higher than that with DM v6.1 (**Table 3**). We compared the transcripts of DMv6.1 and Atlantic genome and found 3729 and 3103 unmatched transcripts, respectively, indicating a high degree of similarity between the transcriptome data and the Atlantic genome. Based on this result, we selected the comparison result with Atlantic in the follow-up analysis.

**Table 3:** Transcriptome data quality and mapping statistics.

Sample	Clean bases (G)	Clean reads	Q20	Q30	GC%	Map for DM (%)	Map for Atlantic (%)
CK1	6.49	43265760	96.32	89.42	42.93	84.93	85.63
CK2	7.21	48081888	96.56	90.18	42.85	85.06	86.06
CK3	6.91	46079524	96.25	89.28	42.82	85.02	86.19
PEG1	8.46	56368200	95.92	88.5	42.75	83.62	85.62
PEG 2	7.21	48052134	96.49	89.87	42.68	84.13	86.02
PEG 3	7.55	50300062	96.23	89.22	42.69	83.99	85.96

CK, control check; PEG, polyethylene glycol treatment group.

### 3.5 Differential expression gene analysis

**Figure 4:**
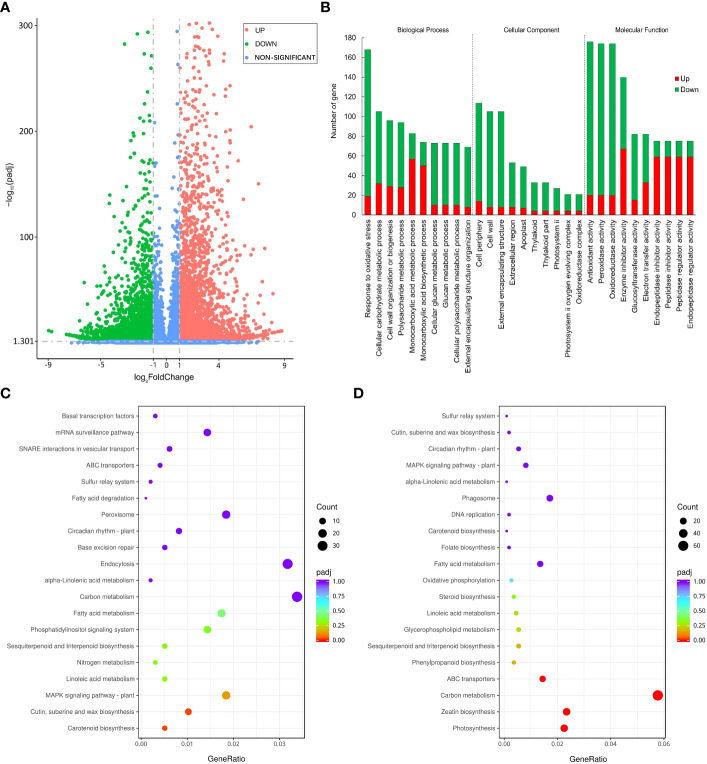
Differentially expressed genes under PEG-induced drought stress in potatoes. **(A)** Volcano plot depicting log_2_FC of up-regulated (red) and down-regulated (blue) genes. **(B)** GO enrichment of differentially expressed genes. **(C)** Top 20 KEGG pathways enrichment of significantly up-regulated genes. **(D)** 20 KEGG pathways enrichment of significantly down-regulated genes.

We used FPKM to evaluate the expression level of genes and used |log_2_(FoldChange)| > 1 & padj < 0.05 as the criteria for screening significantly differentially expressed genes between groups. Finally, a total of 6067 down-regulated differentially expressed genes (DEGs) and 4950 up-regulated DEGs were identified under PEG stress (**Figure 4A**). We performed GO classification and KEGG enrichment on all DEGs and displayed the GO entries with the largest number of genes and the KEGG pathways with the most significant differences in each category. The number of genes responding to oxidative stress in the biological process is the largest, the number of genes in the cell periphery is the largest in the cellular component, and the number of genes with antioxidant activity in the molecular function is the largest (**Figure 4B**). In the scatter diagram of KEGG enrichment, the abscissa is the ratio of the number of differential genes annotated on the KEGG pathway to the total number of differential genes, and the ordinate is the KEGG pathway. The size of the point represents the number of genes annotated on the KEGG pathway, and the color varies from red to purple representing the significant size of the enrichment. Among the up-regulated metabolic pathways, the MAPK signaling pathway, carotenoid biosynthesis, cutin, suberine, and wax biosynthesis had the most significant difference (**Figure 4C**). Among the down-regulated metabolic pathways, carbon metabolism, ABC transporter, zeatin biosynthesis, and photosynthesis were most significantly different (**Figure 4D**). MAPK has been confirmed to positively regulate the modification factors of drought stress, and suberine and wax also play an important role in plant water retention, and their up-regulated expression is conducive to plant response to drought stress. On the contrary, plant growth was inhibited and gene expression related to photosynthesis and material transport was down-regulated under drought stress.

### 3.6 SNP and alternative splicing

**Figure 5:**
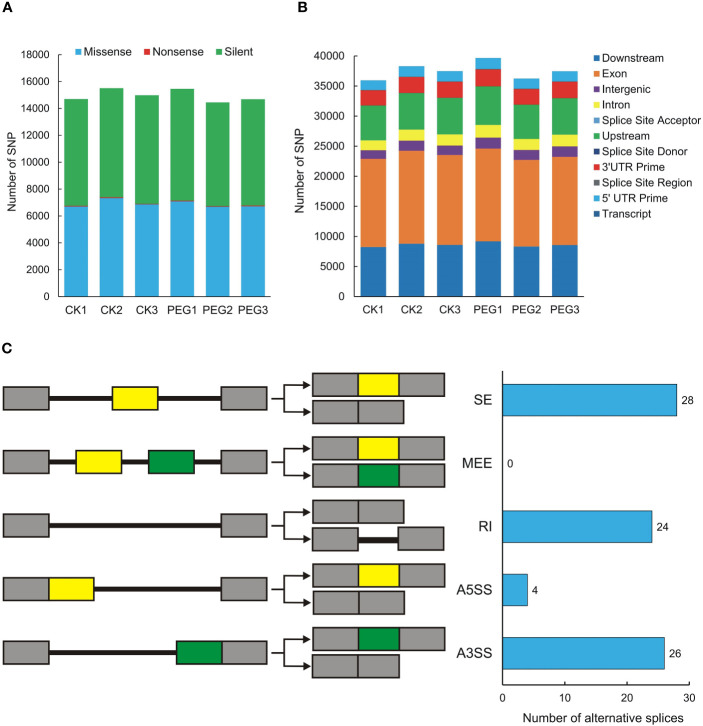
SNPs and alternative splicing of potato DUBs. **(A)** Functional distribution of SNPs. **(B)** Statistics of SNP influence. CK, control check; PEG, polyethylene glycol treatment group. **(C)** Alternative splicing types and numbers of potato DUBs.

Single Nucleotide Polymorphisms (SNP) refers to genetic markers formed by a single nucleotide variation on the genome. We used GATK to detect variant sites and performed statistics based on annotation information. The number of missense and silent was higher in all samples, while the number of nonsense was lower (**Figure 5A**). The SNP region is located in the largest number of exons, followed by downstream and upstream (**Figure 5B**). We also counted the SNPs present in potato DUBs, and the 6 samples contained between 100 and 138 SNPs, all of which were moderate or low impact, and no SNP with high impact was observed (**Table S6**). We used rMATS software for quantitative and differential analysis of Alternative Splicing of potato DUBs. The numbers of Skipped Exon (SE), Alternative 3’ splice site (A3SS), Retained intron (RI), and Alternative 5’ splice site (A5SS) were 28, 26, 24, and 4, respectively. However, a Mutually Exclusive Exon (MEE) in potato DUBs was not observed (**Figure 5C** and **Table S7**).

### 3.7 Expression analysis and interacting proteins of DUBs

**Figure 6:**
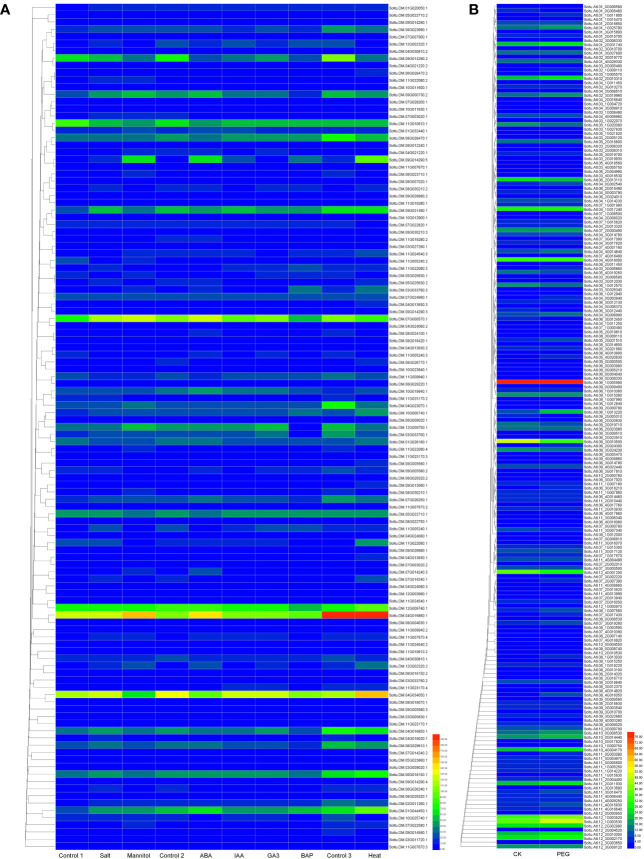
The expression heat map of potato DUBs. **(A)** The expression heat map of DUBs in potato cultivar DM. Control 1 for salt and mannitol, Control 2 for ABA, IAA, GA3, and BAP, and Control 3 for heat. **(B)** Heatmap of expression of DUBs in potato cultivar Atlantic under PEG stress.

**Figure 7:**
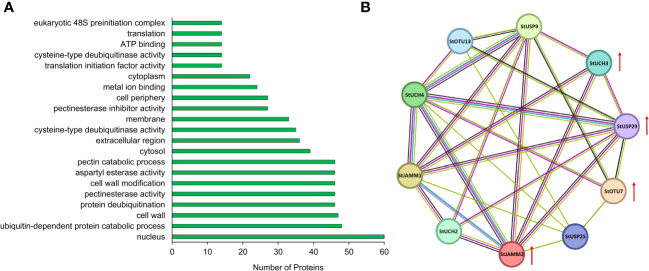
Interacting proteins of potato DUBs. **(A)** GO enrichment of interacting proteins of DUBs. **(B)** DUBs-protein interaction network. Red arrows indicate differentially expressed genes.

We constructed a heat map to compare the expression levels of DUBs genes in DM v6.1 and Atlantic. We found that some DUBs showed the same expression pattern under different treatments, for example, Soltu.DM.08G024100.1, Soltu.DM.12G009740.1, Soltu.DM.12G002320.1 had obvious changes in the expression levels under ABA treatment (**Figure 6A**), and their homologous genes in Atlantic also showed the same trend under PEG stress (**Figure 6B**; **Table S5**). This indicates that DUBs may respond to PEG stress through the ABA signaling pathway. To predict the interacting proteins of all DUBs proteins, we used the STRING software to conduct network interaction analysis based on potato proteins and performed KEGG functional classification of all potential interacting proteins. Most of these interacting proteins were members of the ubiquitination chain, and interestingly, the most interacting proteins localized to the nucleus (**Figure 7A**), suggesting the effect of deubiquitination on gene expression. At the same time, we also found that a large number of DUBs also have interactions (**Figure 7B**; **Table S8**).

### 3.8 Expression profiles of DUBs under drought stress

**Figure 8:**
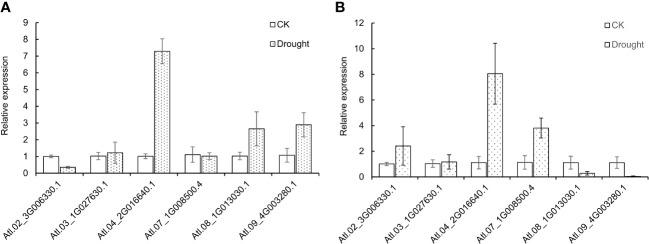
Expression profiles of potato DUBs under drought stress in **(A)** leaves and **(B)** roots.

To further study the expression level of DUBs genes in potatoes under drought stress, 6 DUBs genes were selected from the identified DUBS genes in potato “Atlantic” for the qRT-PCR test. The control and PEG-treated group of invitro “Atlantic” is supplemented in **Figure S3**. Morphological changes in leaves were observed after 12 hours of PEG treatment where the leaves were slightly curled inwards. The results of the qRT-PCR experiment showed that the selected genes showed different expression profiles, but they were roughly consistent with the results of RNAseq. Under drought stress, Soltu.Atl.04_2G016640 was significantly up-regulated in leaves and roots. Some genes had different expression profiles in leaves and roots, for example, Soltu.Atl.02_3G006330.1 was down-regulated in leaves and up-regulated in roots. On the contrary, Soltu.Atl.08_1G013030.1 and Soltu.Atl.09_4G003280.1 were up-regulated in leaves and down-regulated in roots. These results show the complexity of potato DUBs’ response to drought stress (**Figures 8A, B**).

## 4 Discussion

Deubiquitinase is one of the members of the ubiquitination cascade reaction, and it plays an important role in the ubiquitin cycle. DUBs have received less attention than other members of ubiquitin, but this is changing (Zhou et al., 2017). Some members of plant deubiquitinating enzymes have been reported in *Arabidopsis* (Yan et al., 2000), rice (Wang et al., 2018), bamboo (Wu et al., 2019), and wheat (Xu et al., 2021), but there is no related research on the structure and regulatory functions of the potato DUBs family genes. We have previously identified some members of the potato ubiquitination pathway, including UBC (Liu et al., 2019), U-box (Tang et al., 2022), SUMO (Ghimire et al., 2020), etc. We also elucidated that StRFP2 (Qi et al., 2020) and StPUB27 (Tang et al., 2020) are involved in the regulation of drought stress in potatoes. This comprehensive identification of potato DUBs will further improve the research on the potato ubiquitination pathway. Potato has many distantly related species, including diploid wild potato and tetraploid cultivated potato, which have large differences in genetic background. With the availability of the recent tetraploid potato varieties, Atlantic and C88 genomes, we were able to comprehensively search for DUBs in both wild and cultivated potatoes. This allows us to have a more comprehensive picture of potato deubiquitinating enzymes.

We found 75 deubiquitinase genes in DM, 200 in Atlantic, and 302 in C88, and it is clear that the number of deubiquitinase genes is related to the genome size. However, whether it is DM, Atlantic, or C88, their number of deubiquitinating enzymes is not comparable to that of ubiquitin ligases however quantification could determine this (Du et al., 2009). That is to say, the number of deubiquitinating enzymes is significantly less than that of ubiquitin ligases. This indicates that not all ubiquitination of target proteins can be deubiquitinated, and only some of the ubiquitination reactions of proteins are reversible. From the perspective of the interacting proteins of DUBs and their subcellular localization, the target proteins of these DUBs may play a key role in the regulation of cell metabolism, and deubiquitination makes these regulations more flexible. Some scholars believe that monoubiquitination and polyubiquitination do not lead to the degradation of target proteins, but change the activity and function of these proteins, and polyubiquitination is considered to be necessary for ubiquitination degradation (Kwon and Ciechanover, 2017; Swatek et al., 2019). Whether most of the target proteins of deubiquitinating enzymes undergo monoubiquitination and polyubiquitination is not known, but this must be an interesting topic because it indicates that ubiquitination modification regulates the activity and function of target proteins’ flexibility.

Among the 6 types of DUB, the number of USP is the largest, and the number of MJD and MINDY is the least, which shows the same law among the three varieties. USP is also a component that has been studied more in plants, and higher number of its members are reported to be related to the plant growth and stress response (Zhou et al., 2017). We compared the homology of the DUBs of the three species. Atlantic and C88 had more similar DUBs than DM, not only because they were tetraploid, but also because they had a large number of homologous gene-specific expansions. This phenomenon is manifested in their genetic structure and location on chromosomes, as homologous genes tend to cluster together. DUBs also increase the diversity of gene expression products through alternative splicing, which is an effective mechanism to increase the complexity and flexibility of the transcriptome and is a way for plants to adapt to the environment. Alternative splicing plays a key role in potato growth and abiotic stress responses (Laloum et al., 2018; Punzo et al., 2020; Jian et al., 2022). We screened 82 different differential alternative splicing in potato DUBs, including SE, A3SS, RI, and A5SS. These results suggest that potato increases the diversity of DUBs through alternative splicing under PEG stress, thereby enhancing their environmental adaptability.

For the first time, we compared the transcriptome data with the tetraploid potato Atlantic, and the results clearly showed that the accuracy was higher than that with DM, which made our results more reliable. We identified a total of 6067 down-regulated differentially expressed genes (DEGs) and 4950 up-regulated DEGs under PEG stress. MAPK signaling pathway and the synthesis of water-retaining substances such as cutin, suberine, and wax biosynthesis were significantly up-regulated. At the same time, genes related to photosynthesis were significantly down-regulated. These results are consistent with previous research results (Moon et al., 2018; Chen et al., 2019). A large amount of evidence has proved that MAPK is involved in abiotic stress (Danquah et al., 2014; Chen et al., 2021), and the photosynthesis of plants are forced to decline under abiotic stress(Gururani et al., 2015; Kurepin et al., 2015).

DUBs from rice and *Arabidopsis* have been shown to be involved in abiotic stress responses. Arabidopsis UBP12 and UBP13 regulate ABA signaling and drought tolerance by affecting VPS23A ubiquitination (Liu et al., 2022). The NbUBP12 gene in tobacco is involved in the regulation of ABA-mediated stomatal closure, thereby improving drought resistance (Lim et al., 2021). UBP24 can increase the activity of protein phosphatase 2C to enhance ABA sensitivity and salt stress tolerances (Zhao et al., 2016). Knockdown of OsUBP21 expression can increase rice tolerance to heat stress (Zhou et al., 2019). The expression of *Arabidopsis* USP5 and USP13 was also confirmed to be induced by heat stress (Xie et al., 2018). We focused on the expression of DUBs under PEG stress. Among them, 120 DUBs were up-regulated, which was twice the down-regulated expression. However, our previous studies showed that the ubiquitination pathway was also strengthened under PEG stress, which even highlights the specificity of deubiquitinated target proteins. We suggest that under PEG stress, cell metabolism becomes more economical, and it needs to degrade some excess proteins to obtain energy, but at the same time, regulate the function and activity of target proteins through deubiquitination to adapt to environmental changes. This point can be supported by the nuclear localization of interacting proteins of DUBs proteins, because they may be transcription factors that function in the nucleus. DUBs *cis*-acting elements also showed that ABA and ethylene signals can promote their expression, highlighting the close relationship between DUBs and abiotic stress.

In conclusion, we comprehensively characterized DUBs in potato diploid DM and tetraploid Atlantic and C88 and performed the first transcriptome sequencing analysis using Atlantic as a background genome. Our study provides deeper insights into the response of potato deubiquitinating enzymes to osmotic stress, and the rich and precise genetic data package facilitates our genetic studies in tetraploid potatoes.

## 5 Conclusion

In this study, the deubiquitinase family genes of diploid potato DM1-3, tetraploid potato Atlantic and Cooperation-88 were systematically identified, and 75, 117, and 302 members were identified, respectively. The vast majority of deubiquitinase genes are fragmented genes, and there is a large number of alternative splicing phenomena. Potato deubiquitinase gene promoter contains abundant abiotic stress response elements. There are a large number of homologous genes in the DUBs of the three potato varieties, and these homologous genes are often clustered together in the genome. We performed transcriptome sequencing of PEG-induced drought stress in the potato cultivar Atlantic and for the first time analyzed the genome using the Atlantic database as a reference genome. Under PEG stress, a total of 6067 down-regulated differentially expressed genes (DEGs) and 4950 up-regulated DEGs were identified. We focused on the expression of DUBs, 120 DUBs were up-regulated, and most DUBs and their interacting proteins functioned in the nucleus. In conclusion, we conducted a comprehensive identification and analysis of potato DUBs, and accurate transcriptome data will further deepen the understanding of DUBs involved in osmotic stress regulation.

## Data availability statement

The datasets presented in this study can be found in online repositories. The names of the repository/repositories and accession number(s) can be found in the article/**Supplementary Material**.

## Author contributions

XT: Conceptualization, Data curation, Formal Analysis, Funding acquisition, Methodology, Resources, Writing – original draft. FS: Data curation, Formal Analysis, Methodology, Writing – review and editing, Software. NZ: Writing – review and editing, Project administration, Resources, Supervision, Investigation. BR: Writing – review and editing, Validation. RK: Writing – review and editing, Formal Analysis, Visualization. PL: Formal Analysis, Writing – review and editing, Methodology. HS: Writing – review and editing, Funding acquisition, Investigation, Project administration, Resources, Supervision, Validation, Visualization.

## Supplementary material

Supplementary Table 1 The information on the potato deubiquitinase gene.

Supplementary Table 2 The information on potato deubiquitinase.

Supplementary Table 3 The subcellular localization of potato deubiquitinase.

Supplementary Table 4 *Cis*-acting elements of potato deubiquitination genes.

Supplementary Table 5 Orthologs of potato deubiquitination genes.

Supplementary Table 6 Potato deubiquitinase gene SNP.

Supplementary Table 7 Potato deubiquitinase alternative splicing.

Supplementary Table 8 Proteins interacting with StDUBs and differentially expressed under PEG stress.

Supplementary Table 9 qRT-PCR primer information.

Supplementary Figure 1 Phylogenetic tree analysis of potato deubiquitinase genes.

Supplementary Figure 2 Transcriptome data quality assessment. **(A)** Correlation analysis between samples. **(B)** Principal component analysis.

Supplementary Figure 3 The control and 20% PEG 6000 (12h) group of invitro plantlets of potato variety “Atlantic”.
