# Supplementary figures and images for: RNA-seq provides insights into potato deubiquitinase responses to drought stress in seedling stage
Source: Front Plant Sci. 2023 Sep 14;14:1268448. doi: 10.3389/fpls.2023.1268448 (PMC10539648; doi:10.3389/fpls.2023.1268448)

MINDY

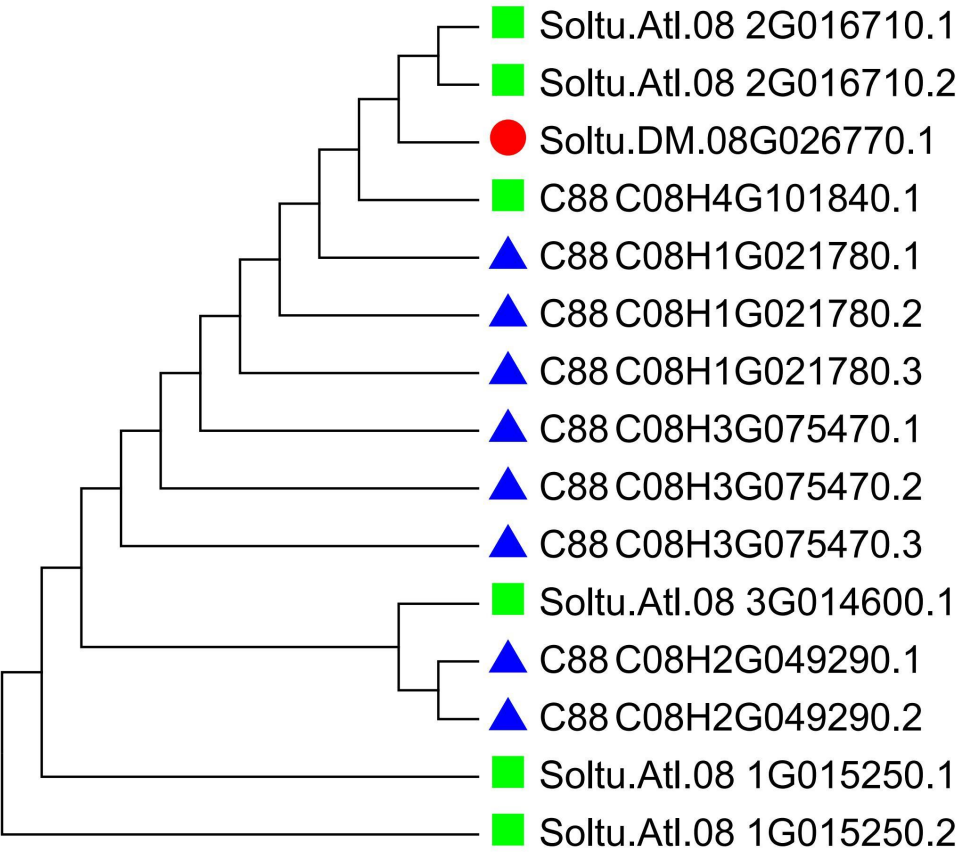

MJD

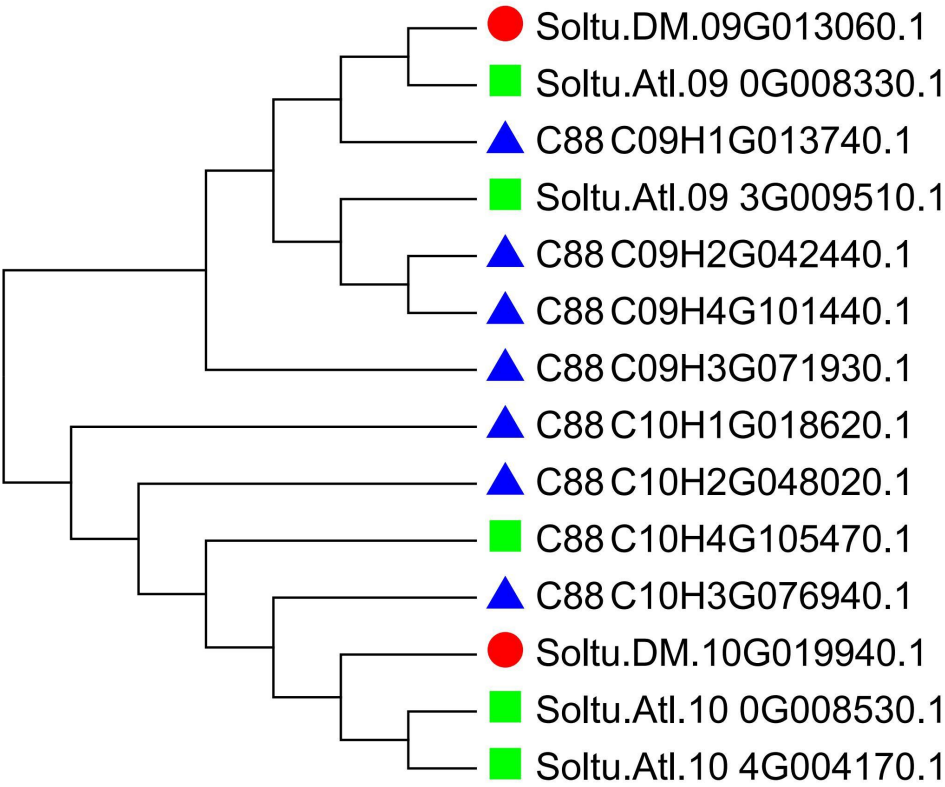

OTU

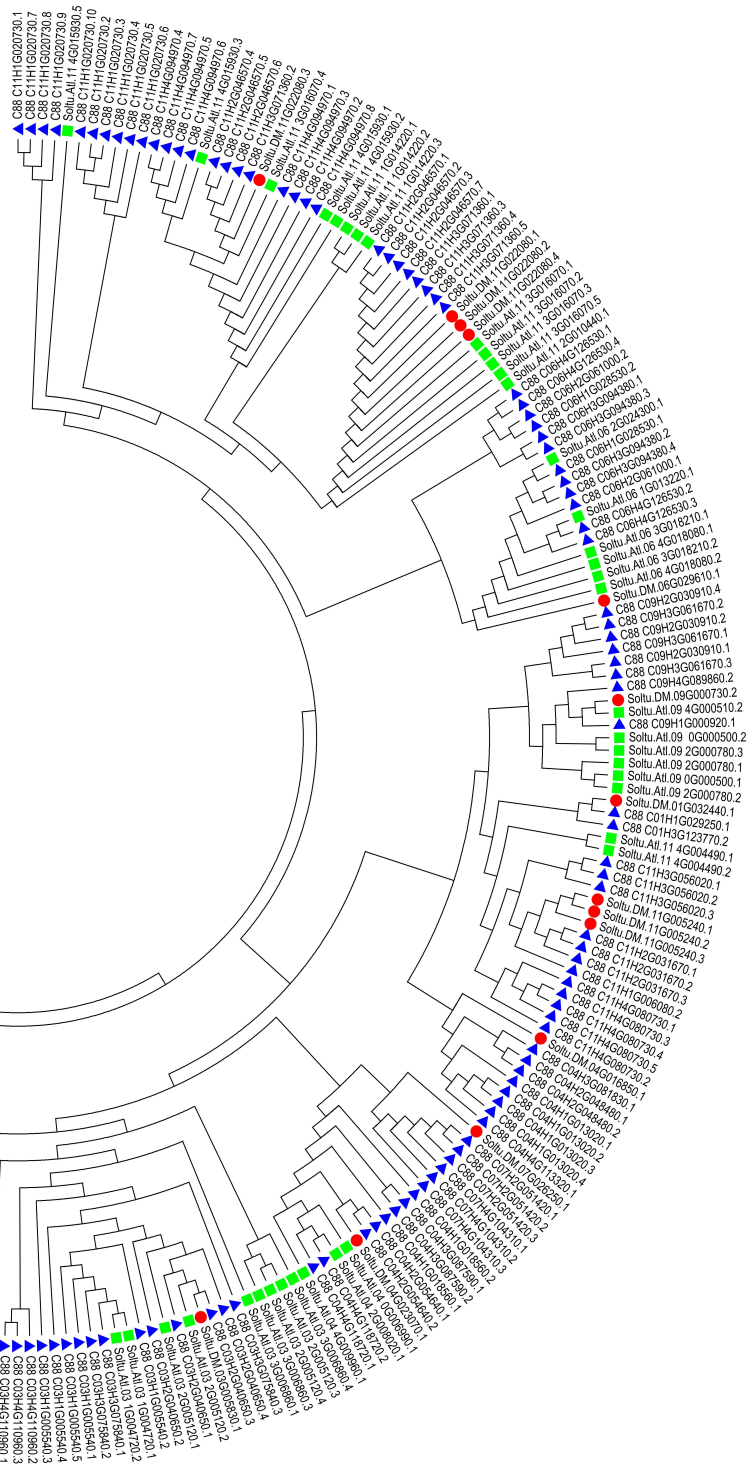

# UCH

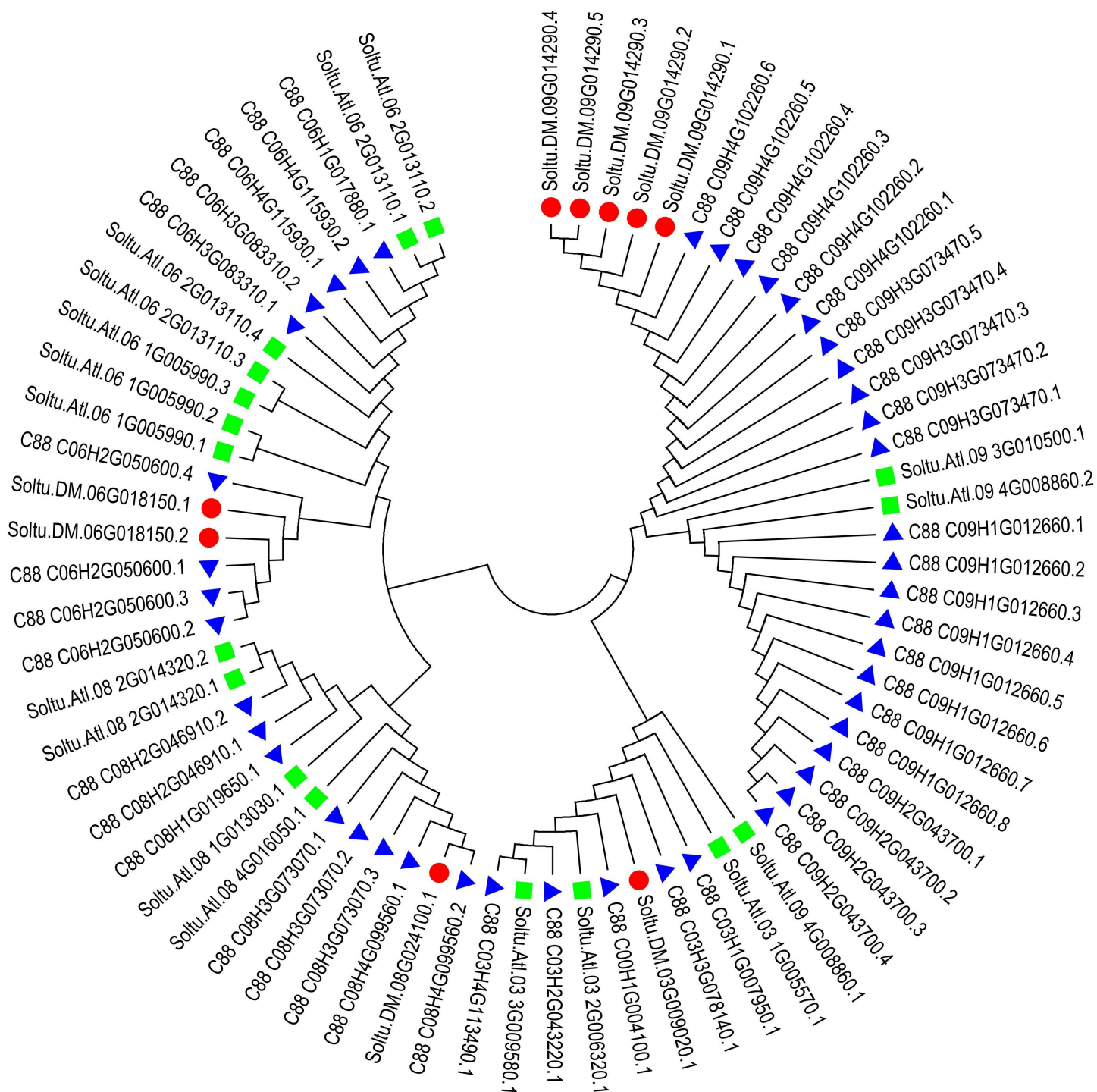

UPS

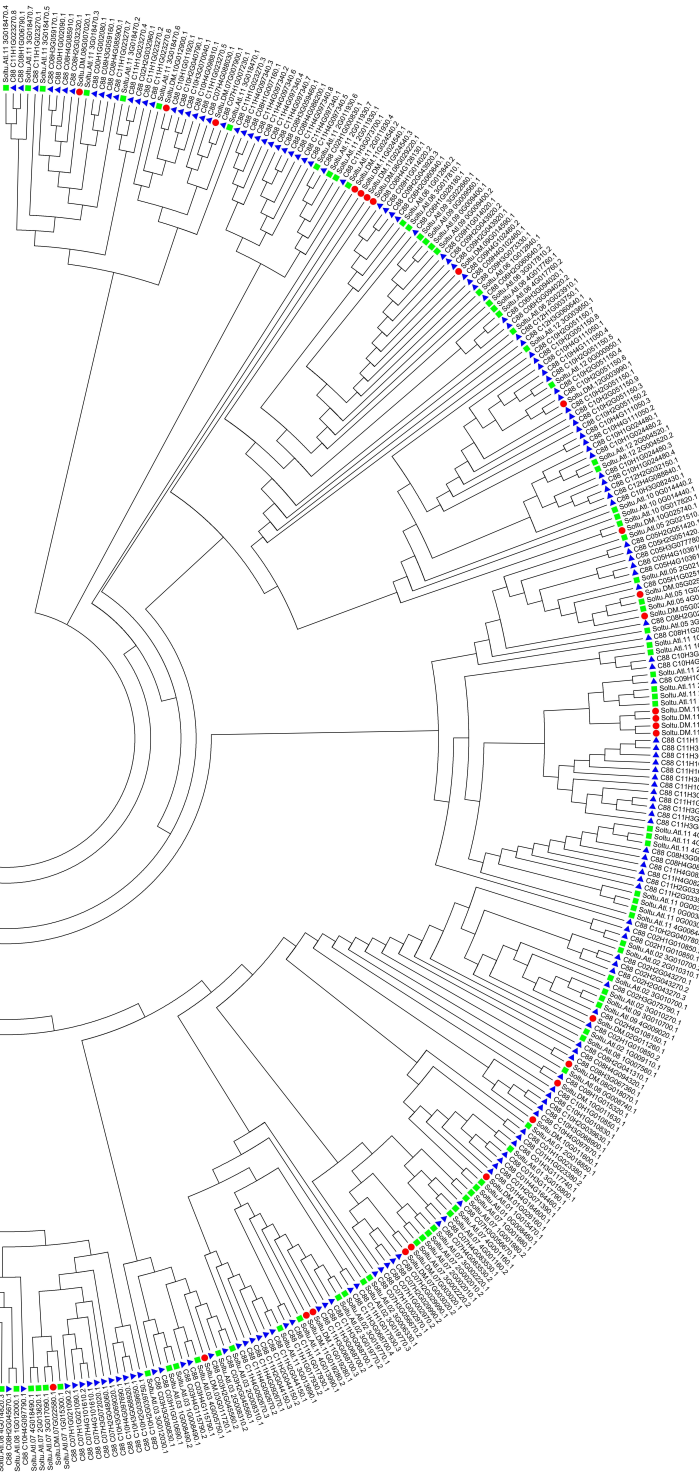

Supplement: Supplementary Table 1 — The information on the potato deubiquitinase gene. [file DataSheet_1.zip › Figure S1.pdf]

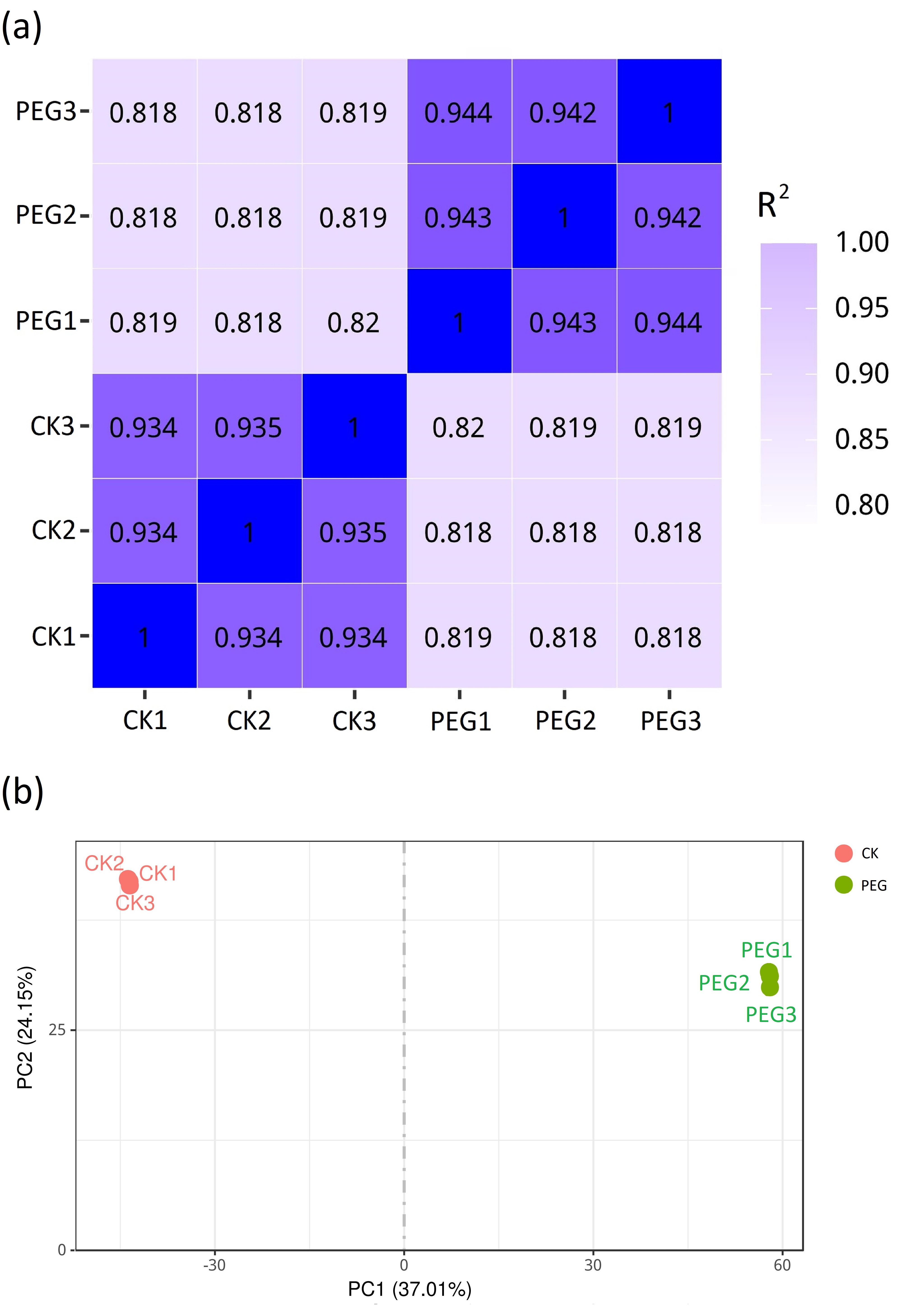

Supplement: Supplementary Table 1 — The information on the potato deubiquitinase gene. [file DataSheet_1.zip › Figure S2.jpg]

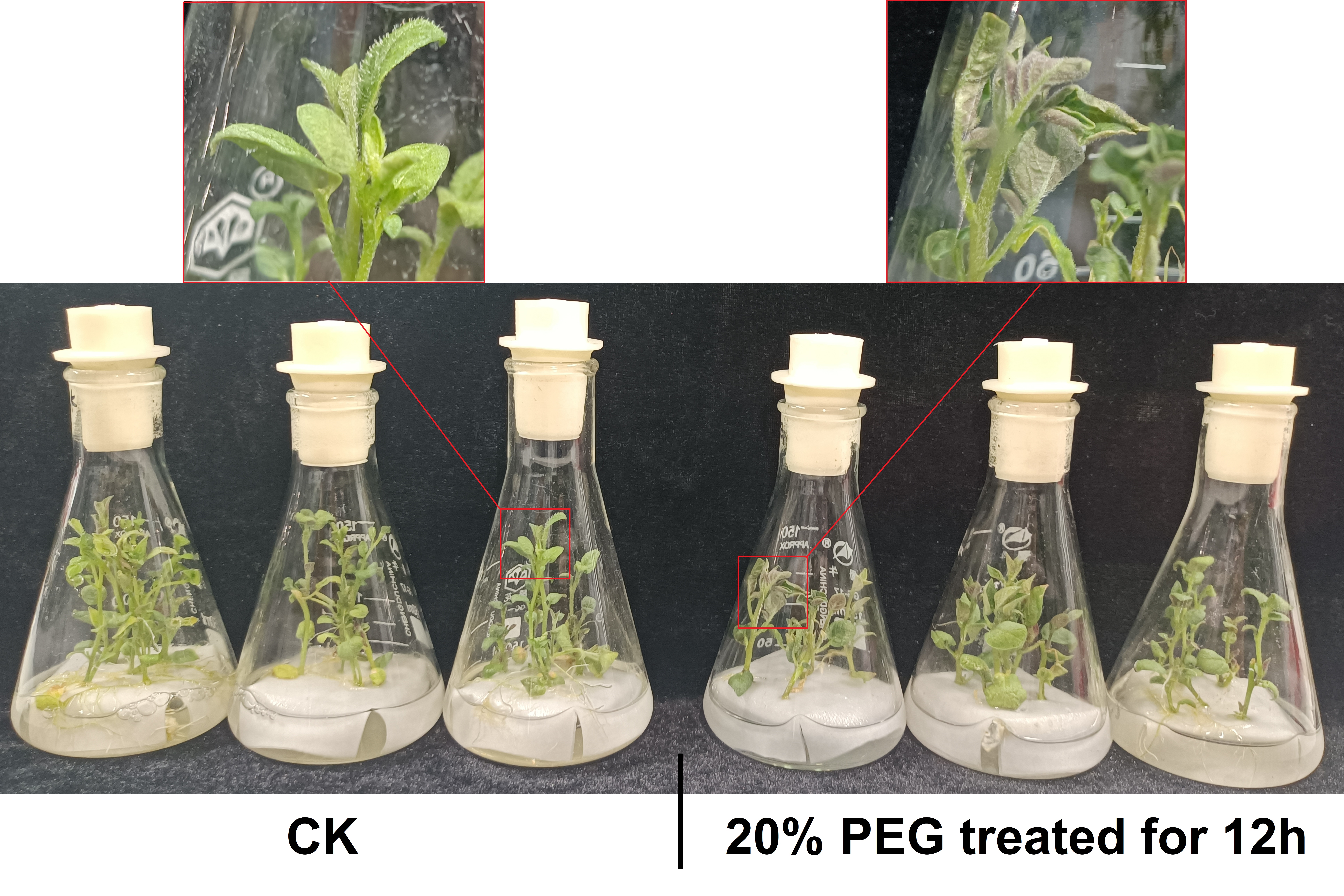

Supplement: Supplementary Table 1 — The information on the potato deubiquitinase gene. [file DataSheet_1.zip › Figure S3.jpg]
